# InCroMAP: integrated analysis of cross-platform microarray and pathway data

**DOI:** 10.1093/bioinformatics/bts709

**Published:** 2012-12-20

**Authors:** Clemens Wrzodek, Johannes Eichner, Finja Büchel, Andreas Zell

**Affiliations:** Center for Bioinformatics Tuebingen (ZBIT), University of Tuebingen, 72076 Tübingen, Germany

## Abstract

**Summary:** Microarrays are commonly used to detect changes in gene expression between different biological samples. For this purpose, many analysis tools have been developed that offer visualization, statistical analysis and more sophisticated analysis methods. Most of these tools are designed specifically for messenger RNA microarrays. However, today, more and more different microarray platforms are available. Changes in DNA methylation, microRNA expression or even protein phosphorylation states can be detected with specialized arrays. For these microarray technologies, the number of available tools is small compared with mRNA analysis tools. Especially, a joint analysis of different microarray platforms that have been used on the same set of biological samples is hardly supported by most microarray analysis tools. Here, we present InCroMAP, a tool for the analysis and visualization of high-level microarray data from individual or multiple different platforms. Currently, InCroMAP supports mRNA, microRNA, DNA methylation and protein modification datasets. Several methods are offered that allow for an integrated analysis of data from those platforms. The available features of InCroMAP range from visualization of DNA methylation data over annotation of microRNA targets and integrated gene set enrichment analysis to a joint visualization of data from all platforms in the context of metabolic or signalling pathways.

**Availability:** InCroMAP is freely available as Java™ application at www.cogsys.cs.uni-tuebingen.de/software/InCroMAP, including a comprehensive user’s guide and example files.

**Contact:** clemens.wrzodek@uni-tuebingen.de or andreas.zell@uni-tuebingen.de

## 1 INTRODUCTION

Typical workflows for the analysis of microarray data involve several steps, namely, the preparation of samples and arrays, their hybridization to arrays, scanning the array and processing the image to read out the raw probe intensities. Depending on the array type, several quality control and low-level data analysis steps are then performed *in silico*. These steps mostly include normalization, annotation of gene identifiers and the calculation of diverse measures of differential probe-level intensities (such as *P*-values, fold changes or log ratios). Mostly, these tasks are performed in R, a statistical programming language (www.r-project.org) or by using derived applications with a graphical user interface (e.g. Mayday; Dietzsch *et al.*, 2006). The processed datasets can then be used in various high-level data analysis tools for further evaluation and data mining. A popular example is the commercial Ingenuity Pathway Analysis software (www.ingenuity.com), which links processed microarray datasets with pathway analysis. However, most of these high-level analysis tools are specialized on single platforms, and only a few approaches are available for an integrated analysis of high-throughput data from heterogenous platforms. Furthermore, not many software tools are freely available that offer suitable and easy-to-use analysis and visualization techniques for microarray platforms, other than mRNA expression arrays.

Therefore, we developed InCroMAP, a user-friendly and interactive application with a graphical user interface that is specialized on an integrated analysis of cross-platform microarray and pathway data. InCroMAP supports DNA methylation, messenger RNA, microRNA and protein modification datasets. Besides these platforms, it is possible to import data from any platform that contains expression values that can somehow be assigned to genes. A special emphasis has been put on the usability of the application. Hence, all required files, for example, for mapping gene identifiers to gene symbols, annotating mRNA targets to microRNAs or pathways to visualize, are either directly included in the application or downloaded dynamically in the background.

## 2 RESULTS

**Fig. 1.**
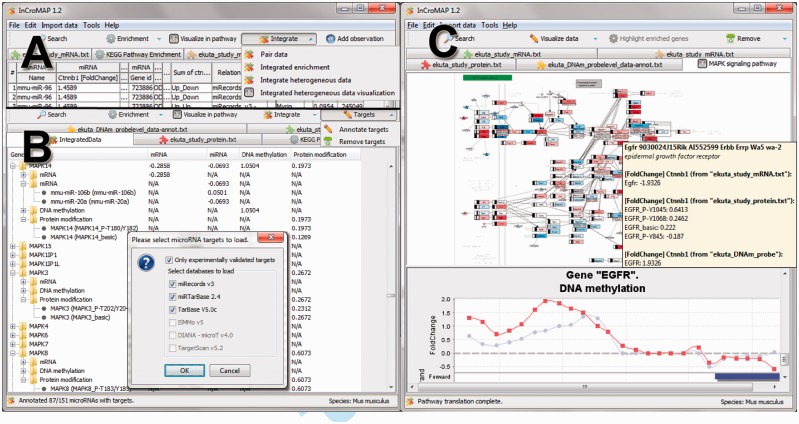
Different views of InCroMAP. (**A**) The pop-up menu shows different methods that are provided for a joint analysis of heterogeneous microarray platforms. (**B**) MicroRNA datasets can be annotated with three experimental and three predicted microRNA target databases directly from within the application. In the background, the result of the ‘integrate heterogeneous data’ procedure is shown. (**C**) Integrated pathway-based visualization of heterogenous microarray datasets allows to visualize up to four different platforms in a single pathway (here: excerpt from the ‘MAPK signalling’ pathway). Pathway nodes can be selected to get more detailed information, including various plots for all assigned expression values (here: DNA methylation in the promoter region of *Egfr*)

To integrate data from multiple platforms, a common denominator must be established. The vast majority of all data are somehow associated with genes. Hence, integration of multiple data types is performed by mapping each probe to a gene. This procedure is straightforward for protein or mRNA datasets. DNA methylation datasets are region based and can be mapped onto genes by defining a window upstream and downstream of each gene’s transcription start site. InCroMAP proposes a window of −2000 and +500 bp as default region, but users may change these values. Integration of microRNA data is performed by annotating the genes of the mRNA targets to each microRNA. For this task, the user can choose between three microRNA target databases that contain experimentally verified targets and three databases with predicted targets (listed in Fig. 1B; databases reviewed in Alexiou *et al.*, 2009).

A first approach to integratively investigate data from any two platforms is the ‘data-pairing’ procedure. This procedure shows two datasets next to each other, thus, simplifying common lookup task, such as investigating the effect of a differentially methylated promoter on mRNA level. Further, this view is especially suitable to inspect the effect of microRNA expression on target mRNAs. An arbitrary amount of data from different platforms can be inspected, using the ‘integrate heterogenous data’ procedure. To keep the clarity, only the most relevant information, that is, the expression values (as fold changes or *P*-values) are shown. Therefore, one row is created for each gene and one column for each platform. A hierarchical representation of the table allows for expanding nodes to get more information, such as all microRNAs targeting this gene’s mRNA (Fig. 1B). A popular method for a generic analysis of expression data is performing a gene set enrichment. We have extended this procedure to an integrated gene set enrichment that is able to perform enrichments across multiple platforms. The user can choose the datasets and thresholds for each dataset to calculate a *P*-value, using a hypergeometric test for each predefined gene set (Backes *et al.*, 2007). InCroMAP supports gene sets from the KEGG PATHWAY database (Kanehisa *et al.*, 2006), Gene Ontology and any gene set from the molecular signatures database (www.broadinstitute.org/gsea/msigdb/). Furthermore, BioPAX Level 2 and Level 3 pathways can be imported for visualization in InCroMAP.

The results of a pathway enrichment can further be visualized in metapathways (e.g. the ‘metabolic pathways’ map), together with mRNA expression data and enriched sub-pathways. All pathways are visualized using KEGGtranslator (Wrzodek *et al.*, 2011), and InCroMAP extends these pathways by visualizing expression data from each single platform therein. Therefore, node colour is changed according to mRNA expression, and small boxes are added and coloured according to each protein modification’s expression value. MicroRNAs are added as small coloured triangles to the graph and are connected to their targets with edges. DNA methylation data are indicated with a black bar that shows the maximum differential peak in each gene’s promoter (stretching from the middle to the left to indicate hypomethylation and to the right for hypermethylation). This is an interactive graph, therefore, allowing users to modify the layout and selecting nodes to get more detailed information and plots of the associated expression data.

Besides those integrated analysis methods, InCroMAP allows plotting region-based DNA methylation data in a genome plot with boxes for gene bodies, which in turn can be coloured according to mRNA expression. Further, all enrichments can also be performed on any single dataset, which is straightforward for mRNA or protein datasets, but implementations that can also handle DNA methylation or microRNA data are less common.
